# Carbon Monoxide Poisoning and Developing Ischemic Heart Disease: A Nationwide Population-Based Nested Case-Control Study

**DOI:** 10.3390/toxics9100239

**Published:** 2021-09-28

**Authors:** Yewon Bahng, Kiook Baek, Jong-Tae Park, Won-Jun Choi, Kyeongmin Kwak

**Affiliations:** 1 Department of Environmental Health Sciences, Graduate School of Public Health, Seoul National University, Seoul 00826, Korea; jupiterwalker@snu.ac.kr; 2 Department of Occupational and Environmental Medicine, Gyeonggi Provincial Medical Center Suwon Hospital, Suwon 16316, Korea; 3 Department of Occupational and Environmental Medicine, Korea University Ansan Hospital, Ansan 15355, Korea; bko8899@gmail.com (K.B.); impjt@korea.ac.kr (J.-T.P.); 4 Department of Occupational and Environmental Medicine, Gil Medical Center, Gachon University College of Medicine, Incheon 21565, Korea; wjchoi@gachon.ac.kr

**Keywords:** carbon monoxide, CO poisoning, CO intoxication, ischemic heart disease, coronary heart disease, cardiac dysfunction, toxic effect, big data, nationwide health database

## Abstract

Although there are several case reports showing that carbon monoxide (CO) poisoning causes ischemic heart disease (IHD), no large-scale epidemiological studies have shown a significant association between the two. To investigate the association between CO poisoning and IHD, a nested case-control study of 28,113 patients who experienced CO poisoning and 28,113 controls matched by sex and age was performed using the nationwide health database of South Korea. Based on a conditional logistic regression, there was a significantly higher risk of IHD among the CO poisoning group than among the control group (adjusted hazard ratio [HR], 2.16; 95% confidence interval [CI], 1.87–2.49). The risk of IHD after CO poisoning was higher among the younger age group under 40 years (adjusted HR, 4.85; 95% CI, 3.20–7.35), and it was much greater among those with comorbidities (adjusted HR, 10.69; 95% CI, 2.41–47.51). The risk of IHD was the highest within the first two years after CO poisoning (adjusted HR, 11.12; 95% CI, 4.54–27.22). Even if more than six years had passed, the risk was still significantly higher than among the control group (adjusted HR, 1.55; 95% CI, 1.27–1.89). The analyses imply that CO poisoning is associated with an increased risk of IHD.

## 1. Introduction

Carbon monoxide (CO) poisoning can occur from brief exposure to CO at excessive levels or from longer exposures at lower levels [1]. The clinical symptoms of CO poisoning are mostly nonspecific and depend on the exposure duration and CO levels [2]. CO poisoning symptoms mainly include neurological symptoms, such as dizziness, nausea, weakness, headaches, lethargy, and confusion [3,4]. CO binds to hemoglobin with a higher affinity than oxygen. By interfering with oxygen binding, it induces tissue hypoxia, which is thought to affect the organs that heavily depend on oxygen utilization, including the cardiovascular system [5]. In a study by Satran et al., 37% of 230 patients with moderate to severe CO poisoning from 1994 to 2002 had a myocardial injury with ischemic electrocardiogram changes and elevated cardiac biomarkers [6,7]. When this patient group was followed up until 2005, the hazard ratio (HR) of long-term mortality was significantly higher in the group with myocardial infarction (MI) at 2.1 (95% confidence interval [CI], 1.2–3.7) compared to the group without MI [8]. Additionally, there have been reports of MI after CO poisoning [9,10,11], and among them, there were cases that occurred at low exposure levels [11]. Therefore, the possibility that the risk of ischemic heart disease (IHD) may increase due to CO poisoning can be considered. However, only a few case reports have confirmed the effects of CO toxicity on the cardiovascular system. Moreover, in a large epidemiological study performed in Taiwan based on a nationwide health database, no increased risk of IHD was found [12]. No large-scale epidemiological studies have explored the relationship between CO poisoning and IHD development other than the study in Taiwan. Therefore, further epidemiological research is needed.

In this study, a nested case-control study was performed using the nationwide health database of South Korea to confirm the association between CO poisoning and IHD risk.

## 2. Materials and Methods

### 2.1. Data Source

The National Health Insurance Database (NHID) of South Korea was used to construct a retrospective cohort for a nested case-control study of data from 2002 to 2017. The NHID was established by the National Health Insurance Service in 2002 and covers the entire Korean population. It contains data on healthcare utilization, sociodemographic variables, health screenings, and mortality [13]. The data on the causes of death, which were provided by Statistics Korea (KoSTAT) and linked to the NHID, were also used.

### 2.2. Study Population

**Figure 1:**
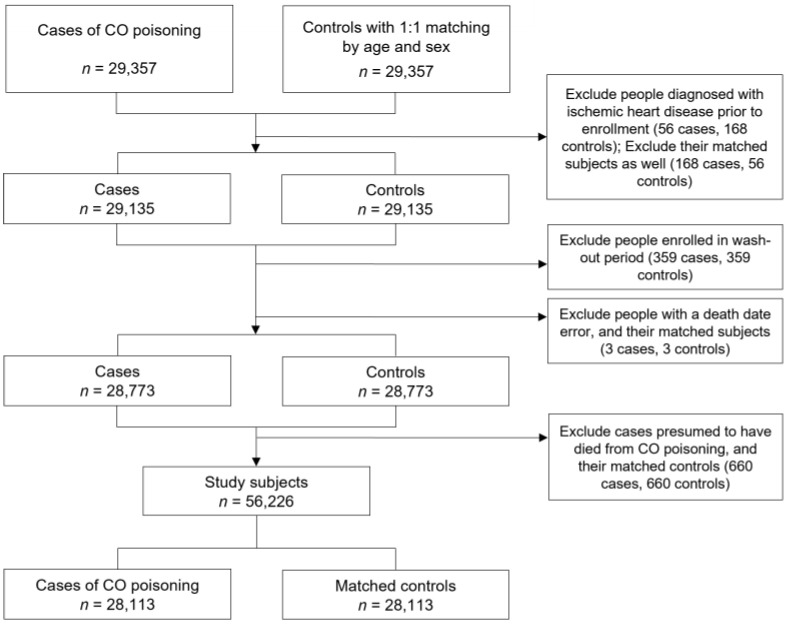
Flowchart for the selection of the study population.

CO poisoning was classified by the International Classification of Diseases, 10th revision (ICD-10) code T58, and all patients treated more than once from 2002 to 2017 were selected as the case group. Controls were also randomly selected among those for whom CO poisoning had not been diagnosed during the same period, and they were matched by sex and age to the case group at a 1:1 ratio. Initially, 29,357 patients and 29,357 controls were included. Among them, 224 cases diagnosed with IHD (I20–I25) prior to enrollment in this study and 224 matched subjects were excluded. Since the NHID was established in 2002 and does not include data from previous years, 2002 was considered a wash-out period, and 359 cases and 359 controls registered in that year were excluded. Additionally, those with errors in the date of death (i.e., those who had medical records after the date of death) were excluded, so three cases and three controls were excluded. Detailed morbidity codes were not provided by KoSTAT because mortality statistics for cases with ICD-10 code T58 are considered sensitive information. Instead, those who died with “toxic effects of substances chiefly nonmedicinal as to source (T51–T65)” within three months of CO poisoning were presumed to have died directly from it. If no information was provided on the cause of death, those who died within two weeks of CO poisoning were presumed to have died from it. These 660 cases of suspected direct death from CO poisoning and 660 matched controls were excluded. Thus, the final study participants consisted of 28,113 CO poisoning cases and 28,113 matched controls (Figure 1).

### 2.3. Definition of Study Outcomes and Comorbidities

Cases of IHD were defined as those who received treatment more than those with ICD-10 codes I20–I25, including at least one inpatient care code. Diabetes mellitus (DM) (E10–E14), hypertension (I10–I15), dyslipidemia (E78), atrial fibrillation (AF) (I48), congestive heart failure (CHF) (I50), hemorrhagic stroke (I60–I62), and ischemic stroke (I63–I69) were defined as comorbidities. DM, hypertension, dyslipidemia, AF, and CHF were diagnosed for patients who received the treatment more than twice with their corresponding ICD-10 codes, while hemorrhagic and ischemic stroke were diagnosed for patients who received the treatment more than once with their corresponding ICD-10 codes.

### 2.4. Covariates

The NHID includes information on socioeconomic factors. Study subject age at the time of enrollment in this study was considered. Residential areas were divided into Seoul, metropolitan cities other than Seoul, and other regions. Household income was classified as high (top 30%), middle, and low (bottom 30%) based on financial contributions to health insurance. Although the NHID’s national health screening database contains information about health behaviors, such as smoking, drinking, and exercise, nearly 40% of the study subjects never had a national health checkup, so much of the information on health behaviors was missing. Information about health behaviors was not used as a covariate because using it with many missing values could affect the results.

### 2.5. Statistical Analysis

Descriptive analyses of sociodemographic characteristics (sex, age, household income, and residential area), comorbidities, and IHD incidence were performed for the CO poisoning and control groups. Chi-square tests were conducted to assess the differences in household income status, the distribution of residential areas, the prevalence of comorbidities, and the incidence of IHD between the CO poisoning and control groups. The incidence rate of IHD was calculated in both the case and control groups, and the incidence rates of CHD according to age group, sex, residential area, household income, and comorbidities were also calculated. Using a conditional logistic regression model, crude and adjusted (i.e., adjusted for residential area, household income, and comorbidities) HRs and their corresponding 95% CIs for IHD development were calculated. Cumulative incidence was also compared between CO poisoning and control groups using a reverse Kaplan–Meier survival curve with a log-rank test [14]. Moreover, crude and adjusted HRs and their corresponding 95% CIs for the CO poisoning and control groups were calculated using stratification according to covariates and follow-up periods. All statistical analyses were conducted using SAS version 9.4 (SAS Institute, Cary, NC, USA).

## 3. Results

### 3.1. Baseline Characteristics of the Subjects

The average age of the study subjects was 37.4 years. Most subjects (36.4%) were aged 40–65 years, followed by those aged 25–39 years (32.9%), <25 years (23.5%), and ≥65 years (7.2%). There were more men (54.8%) than women. The sex and age distributions among the CO poisoning and control groups were matched. As for the residential area, the proportion of subjects living in Seoul was higher among the control group (20.4%), while the proportion of subjects living in areas other than metropolitan cities, including Seoul, was higher among the CO poisoning group (59.6%). As for the household income level, the proportion of subjects belonging to the high-income group was higher among the control group (36.2%), while the proportion of subjects belonging to the low-income group was higher among the CO poisoning group (31.6%). When considering comorbidities, there were no significant differences in the prevalence of hypertension and AF between the CO poisoning and control groups. The prevalence rates of DM, dyslipidemia, CHF, and hemorrhagic stroke were significantly higher in the CO poisoning group, while the prevalence of ischemic stroke was significantly higher in the control group (Table 1).

### 3.2. Risk of Developing Ischemic Heart Disease

**Figure 2:**
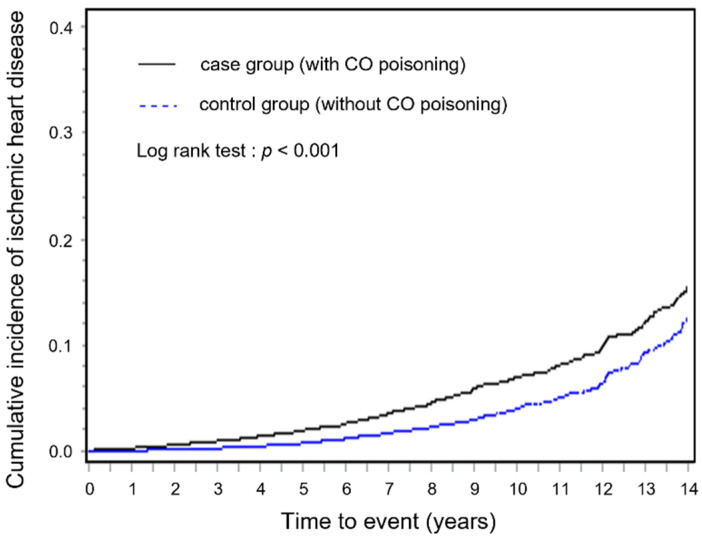
Comparison of cumulative incidence rates among the CO poisoning and control groups.

From 2003 to 2017, the number of cases with IHD among the CO poisoning and control groups were 729 and 386, respectively. The risk of developing IHD was significantly higher for the CO poisoning group than the control group by approximately 2.2 times (crude HR, 2.19; 95% CI, 1.91–2.51). The risk was significantly higher for the case group than in control group, even after adjusting for residential area, household income, and the presence or absence of comorbidities (adjusted HR, 2.16; 95% CI, 1.87–2.49) (Table 2). The reverse Kaplan–Meier survival curves showed that the cumulative incidence of IHD over time was consistently significantly higher among the CO poisoning group than among the control group (log-rank test: *p* < 0.001; Figure 2).

When analyzed by stratification according to socioeconomic factors, the risk of developing IHD among the CO poisoning group compared with the control group was similar in men and women. The risk of developing IHD among the CO poisoning group was much higher for the younger age group (<40 years) (adjusted HR, 4.85; 95% CI, 3.20–7.35), and no significantly increased risk was found for patients aged ≥65 years (adjusted HR, 1.15; 95% CI, 0.87–1.51). The risk of developing IHD among the CO poisoning group was the highest for those living in metropolitan areas, except Seoul (adjusted HR, 3.69; 95% CI, 1.79–7.61), while those living in Seoul did not show any significant increase in risk (adjusted HR, 1.25; 95% CI, 0.53–2.95). Based on household income levels, there was a significantly increased risk of developing IHD among the CO poisoning group for all household income deciles, but the effect size of the risk for the high-income group was relatively low. When stratified according to comorbidities, the risk of developing IHD among the CO poisoning group was higher for those with comorbidities (crude HR, 3.00; 95% CI, 1.67–5.38), and this risk was even greater when the model adjusted for covariates (adjusted HR, 10.69; 95% CI, 2.41–47.51) (Table 3).

When stratified according to follow-up periods, the risk of developing IHD associated with CO poisoning was increased by approximately 5.2 times within two years of CO poisoning (crude HR, 5.24; 95% CI, 3.12–8.80), and this risk was much greater—by 11 times—after the model adjustment for other covariates (adjusted HR, 11.12; 95% CI, 4.54–27.22). After more than two years of CO poisoning, the risk of developing IHD decreased, but it showed a consistent and significant increase when compared to the control group. Even after ≥ 6 years, the risk was increased by ≥ 1.5 times (adjusted HR, 1.55; 95% CI, 1.27–1.89) (Table 4).

## 4. Discussion

The effect of CO poisoning on the development of IHD was analyzed using data from the nationwide health database of South Korea. The results indicate that there is a significant association between CO poisoning and an increased risk of developing IHD. This study excluded patients who died directly from CO poisoning and confirmed that the long-term risk of IHD was significantly elevated. In particular, the level of risk was high in the younger age group, and it was confirmed that the risk of developing IHD was much greater in the presence of comorbidities related to IHD.

Only limited studies have been conducted to evaluate the associations between CO poisoning and the risk of developing IHD. Although subsequent MIs have been reported several times after CO poisoning has occurred [9,10,11], it is not known whether the risk increases with time. The risk of ischemic stroke in the CO poisoning group was previously analyzed using the NHID. The results showed a significant 2.3-fold increase in risk, similar to the results of this study [15]. Ischemic stroke and IHD have common risk factors, and there are some similarities in the mechanisms of their development. Therefore, it is reasonable to approach IHD development caused by CO poisoning based on the common mechanism of ischemic stroke and IHD. Both ischemic stroke and IHD have atherothrombosis as an etiological factor. Exposure to CO enhances coagulation by binding to a fibrinogen-bound heme, which may lead to thromboembolism [16,17,18]. Several epidemiological studies and case reports have confirmed the prothrombic potential of CO exposure to IHD [9,10,19,20]. Thromboembolism not only directly causes IHD but may also increase the long-term risk of developing IHD. A study using Taiwan’s nationwide health database also found a significantly increased long-term risk of cardiovascular events, including IHD, in patients with thromboembolism [21]. This CO-induced thromboembolism is the main mechanism that causes IHD in the early stages, and it can also increase the long-term risk of developing IHD, although this risk decreases with time.

The nationwide health database in Taiwan was also used to analyze the risk of cardiovascular disease, including IHD, in patients with CO poisoning from 2000 to 2011 [12]. The analysis revealed an elevated risk of developing IHD among patients with CO poisoning, with an adjusted HR of 1.14. This elevated risk was not significant (95% CI, 0.93–1.40). However, considering that the HR was significantly higher at 1.75 (95% CI, 1.06–2.89) for the group with severe CO poisoning and that the risk of IHD occurrence within 3 years was significant (HR, 1.33; 95% CI, 1.00–1.76), the results of the Taiwanese study cannot rule out increased IHD risk due to CO poisoning. Various reasons for differing results between our study and the Taiwanese study can be considered. The population of South Korea is more than twice that of Taiwan, and our study had a slightly longer follow-up period than the Taiwanese study; therefore, the number of patients with CO poisoning was much greater in our study. In addition, our study performed 1:1 matching, and the Taiwanese study matched four-fold controls; thus, the composition of the control group may be different. Moreover, we used conditional logistic regression because our study was designed as a nested case-control study [22,23], but the study in Taiwan was analyzed using Cox’s proportional hazard regression model. It is thought that these differences in the number of patients, control group composition, and statistical methods may have yielded different results. In addition to these demographic and methodological differences, other factors potentially influencing the occurrence of IHD may differ, further yielding differing outcomes. Lastly, clinical case management after CO poisoning may be different, allowing for different effects on IHD risk. However, no difference in CO poisoning case management was confirmed because this information was not provided by the nationwide health database.

Another mechanism by which CO poisoning may increase the risk of IHD is through the development of arrhythmias, including AF. Hypoxia and cardiac dysfunction caused by CO poisoning can induce paroxysmal tachycardia, paroxysmal ventricular tachycardia, ventricular fibrillation or flutter, and atrial fibrillation or flutter [12,24]. AF is known to be a risk factor for IHD and ischemic stroke [25,26]. It was hypothesized that the induction of arrhythmias, including AF, may play a role in increasing the long-term risk of IHD.

In this study, more than 60% of cases of CO poisoning occurred in younger patients under 40 years of age. The risk of developing IHD after experiencing CO poisoning in the younger age group (<40 years) was much higher than that in other age groups. The higher the age group, the lower the risk of IHD after CO poisoning. In our analysis, no significant increase in risk was found in patients over 65 years of age. While there are several risk factors related to the occurrence of IHD in the elderly group, the incidence of IHD in the younger age group was not high, and the related risk factors were less prevalent among the younger age group than in the elderly group, which was thought to be due to the strong effect of CO exposure.

Regarding comorbidities, patients with underlying diseases related to IHD, such as hypertension, DM, dyslipidemia, CHF, AF, and stroke, had a higher risk of developing IHD after CO poisoning than those without. When the residence and income levels were adjusted, it was confirmed that the risk in the presence of comorbidities was significantly higher than that in the control group. This suggests that hypoxemia, cardiac dysfunction, and thromboembolism after CO exposure more sensitively affect people with underlying heart disease-related comorbidities, which may increase the risk of developing IHD.

This study has several limitations. First, there are questions about the validity of the diagnosis of IHD. Because the NHID contains claims data, there are cases where a diagnosis code was only entered for the purpose of a claim, and diagnostic validity based on the ICD code is always doubtful because of these factors. Here, an operational definition of the disease was made to correct for these limitations, but we could not completely solve this problem. In addition, the NHID did not include radiographic images or laboratory data, and the information about health behaviors was not utilized because nearly 40% of the subjects’ information was missing. There are limitations among the complete retrospective cohort due to the insufficient information provided for clinical records and health behaviors. Next, although the controls were randomly selected through the matching of their sex and age at a 1:1 ratio, there is a possibility that the case and control groups for the risk factors of IHD are not uniform, so bias might occur. Finally, the number of CO poisoning cases was relatively small before 2010, even if the recent increase in CO poisoning cases was considered. There is a high possibility that CO poisoning cases were omitted from the NHID before 2010, and it is possible that the results may be biased due to such omissions.

Despite these limitations, this study is meaningful as it is a retrospective study built on all reported cases of CO poisoning in South Korea from 2002 to 2017. Another strength is that the comparability was improved by selecting controls that were matched by sex and age. Additionally, it was appropriate to explore the long-term cardiogenic effect by linking the data on causes of death from KoSTAT, excluding subjects who were presumed to have died directly from CO poisoning.

## 5. Conclusions

Our study showed an association between CO poisoning and increased IHD risk. IHD risk was the highest within 2 years after CO poisoning. Although risk decreased over time, it remained significant for 6 years or more, implying that people who experience CO poisoning might have a substantial long-term risk of developing IHD. This increased risk was more pronounced among the younger age group and the group with comorbid diseases. Although we observed increased IHD risk after CO poisoning, the exact mechanism of this association remains unclear. Further studies of the long-term effects of CO poisoning on the cardiovascular system and the underlying mechanisms are thus required.

## Figures and Tables

**Table 1:** General characteristics of the subjects.

Characteristics	Carbon Monoxide Poisoning *n* = 28,113	Control *n* = 28,113	*p*-Value
Mean age (years) *	37.4 ± 17.6	37.4 ± 17.6	1.000
Age (years) ^†^			1.000
<25	6600 (23.5%)	6600 (23.5%)	
25–39	9429 (32.9%)	9429 (32.9%)	
40–64	10,247 (36.4%)	10,247 (36.4%)	
≥65	2017 (7.2%)	2017 (7.2%)	
Sex ^†^			1.000
Male	15,417 (54.8%)	15,417 (54.8%)	
Female	12,696 (45.2%)	12,696 (45.2%)	
Residential area ^†^			<0.001
Seoul	4616 (16.4%)	5738 (20.4%)	
Other metropolitan cities	6745 (24.0%)	6841 (24.3%)	
Other areas	16,752 (59.6%)	15,534 (55.3%)	
Household income ^†^			<0.001
High	8192 (29.1%)	10,169 (36.2%)	
Middle	11,042 (39.4%)	10,650 (37.9%)	
Low	8879 (31.6%)	7294 (25.9%)	
Comorbidity ^†^			
Hypertension	2314 (8.3%)	2386 (8.5%)	0.273
Diabetes	2223 (7.9%)	1788 (6.4%)	<0.001
Dyslipidemia	4875 (17.3%)	4045 (14.4%)	<0.001
Atrial fibrillation	127 (0.2%)	69 (0.3%)	0.329
Congestive heart disease	187 (0.7%)	95 (0.3%)	<0.001
Hemorrhagic stroke	259 (0.9%)	77 (0.3%)	<0.001
Ischemic stroke	329 (1.2%)	431 (1.5%)	<0.001

* Values are presented as mean ± standard deviation. ^†^ Values are presented as number of subjects (%).

**Table 2:** Incidence rates and hazard ratios for ischemic heart disease among the CO poisoning and control groups.

Groups	No. of Cases	Person-Years	Incidence, per 1000 Person-Years	HR * (95% CI)	
				Crude	Adjusted ^†^
Control	386	125,466.7	3.08	1.00	1.00
Carbon monoxide poisoning	729	128,860.3	5.66	2.19 (1.91–2.51)	2.16 (1.87–2.49)

* HR, hazard ratio. ^†^ Adjusted for residential area, household income, and presence or absence of comorbidities.

**Table 3:** Incidence rates and hazard ratios of ischemic heart disease as stratified by sex, age, residential area, household income, and comorbidities.

Variables	Carbon Monoxide Poisoning			Control			HR * (95% CI) ^†^	
	No. of Cases	Person-Years	Incidence	No. of Cases	Person-Years	Incidence	Crude	Adjusted ^‡^
Sex								
Male	417	65,330.5	6.38	217	67,597.7	3.21	2.24 (1.86–2.67)	2.18 (1.81–2.63)
Female	312	60,136.2	5.19	169	61,262.6	2.76	2.15 (1.74–2.65)	2.17 (1.73–2.71)
Age (years)								
<40	166	71,042.7	2.34	38	72,273.6	0.53	4.69 (3.25–6.75)	4.85 (3.20–7.35)
40–64	371	44,444.6	8.35	175	45,992.6	3.80	2.37 (1.95–2.87)	2.27 (1.85–2.78)
≥65	192	9979.4	19.24	173	10,613.2	16.30	1.28 (0.97–1.58)	1.15 (0.87–1.51)
Residential area								
Seoul	88	21,793.9	4.04	80	31,035.1	2.58	1.33 (0.63–2.82)	1.25 (0.53–2.95)
Other metropolitan cities	143	28,120.8	5.09	86	32,434.9	2.65	3.33 (1.75–6.35)	3.69 (1.79–7.61)
Other areas	498	75,552.0	6.59	220	65,390.2	3.36	2.09 (1.67–2.60)	2.15 (1.70–2.72)
House income								
High	196	36,389.4	5.39	150	46,260.0	3.24	1.90 (1.29–2.80)	1.99 (1.30–3.05)
Middle	270	48,954.8	5.52	125	47,343.6	2.64	2.69 (1.82–3.96)	2.75 (1.82–4.15)
Low	263	40,122.5	6.55	111	35,256.7	3.15	2.83 (1.76–4.55)	2.64 (1.58–4.38)
Comorbidity								
No	520	105,695.0	4.92	300	110,784.5	2.71	2.12 (1.78–2.53)	2.05 (1.73–2.44)
Yes	209	19,717.7	10.57	86	18,075.9	4.76	3.00 (1.67–5.38)	10.69 (2.41–47.51)

* HR, hazard ratio. ^†^ The HR indicates the level of risk among the CO poisoning group compared with that of the control group. ^‡^ Adjusted for residential area, household income, and the presence or absence of comorbidities.

**Table 4:** Incidence and hazard ratios of ischemic heart disease as stratified by follow-up period.

Follow-Up Periods	Carbon Monoxide Poisoning			Control			HR * (95% CI) ^†^	
	No. of Cases	Person-Years	Incidence	No. of Cases	Person-Years	Incidence	Crude	Adjusted ^‡^
≤2	130	7747.3	16.78	19	7372.4	2.58	5.24 (3.12–8.80)	11.12 (4.54–27.22)
2–4	131	22,208.7	5.90	59	22,273.5	2.65	2.13 (1.51–3.00)	2.15 (1.44–3.20)
4–6	122	25,064.0	4.87	71	25,985.3	2.73	2.23 (1.58–3.15)	2.72 (1.80–4.13)
>6	346	70,446.6	4.91	237	73,229.1	3.24	1.64 (1.36–1.98)	1.55 (1.27–1.89)

* HR, hazard ratio. ^†^ The HR indicates the level of risk among the CO poisoning group compared with that of the control group. ^‡^ Adjusted for residential area, household income, and the presence or absence of comorbidities.

## Data Availability

The data used in this study are available from the Korean National Health Insurance Service (KNHIS). However, these data require deliberation and approval by the KNHIS and can be assessed only through the local intra-network of the KNHIS.
